# The Gut-Brain Axis and Mental Health: How Diet Shapes Our Cognitive and Emotional Well-Being

**DOI:** 10.7759/cureus.88420

**Published:** 2025-07-21

**Authors:** Shradha Patil, SayedFarhan S Mehdi

**Affiliations:** 1 Trauma and Orthopaedics, Diana, Princess of Wales Hospital, Grimsby, GBR; 2 Plastic Surgery, Bradford Royal Infirmary (Bradford Teaching Hospitals NHS Foundation), Bradford, GBR

**Keywords:** diet, gastrointestinal microbiome, gut-brain axis, mental health, nutrition, probiotics

## Abstract

The gut-brain axis (GBA) connects the gastrointestinal (GI) system and the central nervous system (CNS) in a two-way communication system that greatly impacts mental health and overall well-being. Dietary choices significantly influence the gut microbiome, thereby affecting emotional, cognitive, and neurological health. This review explores how specific dietary patterns, including high-fiber, plant-based, and Mediterranean diets (MD), enhance microbial diversity, decrease inflammation, and enhance gut-brain communication. It highlights the importance of probiotics, prebiotics, omega-3 fatty acids, and antioxidants in controlling gut microbiota, enhancing intestinal barrier function, and reducing systemic inflammation associated with anxiety, depression, and cognitive decline. The article covers practical methods for integrating dietary adjustments into personalized mental health treatment, while also addressing challenges such as individual microbiome variability and the need for long-term research. This review underscores the possibility of using nutritional interventions as a non-invasive and easily accessible way to promote mental health and resilience based on existing evidence. The methodology involved a comprehensive examination of existing literature exploring the GBA and its influence on mental health. We searched relevant databases, including PubMed and Google Scholar, using keywords such as "gut-brain axis," "microbiome," "diet," and "mental health." Peer-reviewed articles were selected based on their relevance to the relationship between dietary interventions and mental health outcomes, with a focus on studies exploring cognitive and emotional well-being.

## Introduction and background

The gut-brain axis (GBA), a complex bidirectional communication network between the gastrointestinal (GI) tract and the central nervous system (CNS), plays a significant role in maintaining both physiological and psychological health [1,2]. This communication is facilitated through neural, hormonal, and immune pathways, and emerging evidence suggests that the gut microbiota, a diverse ecosystem of microorganisms in the gut, may influence this axis [3]. Often termed the 'second brain,' the microbiota has been shown in some studies to influence the CNS through pathways such as neurotransmitter production and immune modulation [4]. This interaction affects mood, cognition, and emotional responses, indicating the microbiota’s far-reaching influence on mental health, though much remains to be understood [5]. Imbalances in microbial communities (dysbiosis) may disrupt this axis, potentially leading to inflammation and neurotransmitter disturbances that have been implicated in conditions like depression and anxiety [6,7]. Studies have demonstrated that gut-derived metabolites, such as short-chain fatty acids (SCFAs), which are produced by gut bacteria when digesting fiber, and tryptophan derivatives, which are compounds involved in the production of serotonin, influence brain function by crossing the blood-brain barrier or modulating immune responses [8]. These findings have sparked interest in the possibility that microbiome imbalances may contribute to the development or severity of mental health conditions [9].

This growing body of research has raised interest in microbiome-targeted approaches, such as probiotics, prebiotics, and dietary changes, as potential adjunctive strategies for improving mental well-being [2,7]. Certain probiotics, known as psychobiotics, have been shown to produce mood-regulating neurotransmitters like serotonin and gamma-aminobutyric acid (GABA), which are crucial for managing stress and anxiety [7,10]. This could be particularly relevant in low- and middle-income countries, where mental health services are scarce and affordable alternatives are urgently needed [9]. In such settings, dietary modifications and probiotic treatments offer an accessible and cost-effective approach to mental health care [1]. While animal studies have underscored the influence of microbiota on neurodevelopment, human studies suggest that dietary choices, such as consuming fiber-rich or fermented foods, can promote beneficial gut bacteria that may enhance mood and cognition [9,6]. Although these findings are promising, many studies remain preliminary, and further research is needed to explore individualized dietary and microbial interventions based on unique microbiome compositions [5]. This review consolidates current findings on how dietary interventions may impact mental health through the GBA, emphasizing the need for continued exploration into the role of the microbiome as a therapeutic target for mental well-being [3]. This is a narrative review aimed at summarizing current evidence on the GBA, with a focus on microbial metabolites, dietary influences, and mental health. It does not follow the methodology for systematic reviews.

## Review

Methodology

Relevant peer-reviewed literature was selected through searches of databases such as PubMed and Google Scholar using terms including "gut-brain axis," "microbiome and mental health," "psychobiotics," "probiotics," and "dietary interventions," focusing on studies published in the last 10-15 years.

The role of the gut-brain axis in mental health

The GBA is a complex communication network linking the GI system and CNS through neural, hormonal, immune, and metabolic pathways [9]. The influence of the GBA on mental health has become more evident, with research highlighting the role of the gut microbiota: a diverse ecosystem of microorganisms within the GI tract that produces neurotransmitters, hormones, and metabolites that play key roles in brain function [10,11]. The vagus nerve, one of the primary pathways between the gut and the brain, facilitates direct communication that can modulate brain activity and influence behaviors associated with anxiety and mood [12]. Dysbiosis, or an imbalance in the gut microbiota, has been closely linked to neuropsychiatric disorders such as anxiety, depression, and cognitive impairment, as the altered microbiota can disrupt normal signaling along the GBA [13,14].

Disruption of gut integrity, or "leaky gut," is another outcome of dysbiosis, where increased intestinal permeability allows pro-inflammatory molecules to enter circulation, cross into the bloodstream, and ultimately pass through the blood-brain barrier (BBB) [15]. This can result in neuroinflammation, which is known to exacerbate symptoms of mood disorders by interfering with normal neural function [16]. Furthermore, chronic dysbiosis has been shown to interfere with brain plasticity, reducing the brain’s ability to adapt to stress and environmental changes and limiting resilience to mental health challenges [17,18].

Microbial metabolites, particularly SCFAs like butyrate, acetate, and propionate, are essential in reinforcing the gut barrier, supporting anti-inflammatory pathways, and regulating the production of neurotransmitters such as serotonin and GABA, both of which are vital for emotional stability [19,20]. Approximately 90% of serotonin is synthesized in the gut under microbial influence, underscoring the microbiota’s critical role in mental health [21,22]. While emerging studies suggest that specific probiotic strains, including *Lactobacillus *and *Bifidobacterium*, may influence the body's stress response system via the vagus nerve and the hypothalamic-pituitary-adrenal (HPA) axis, more research is needed to confirm these mechanisms [22].

Psychobiotics, which include specific probiotics and prebiotics known for their mental health benefits, have demonstrated potential in easing symptoms of anxiety, depression, and stress-related disorders by balancing gut microbiota, decreasing neuroinflammation, and modulating neurotransmitter activity [23-25].

How diet shapes the gut microbiome

Diet is a foundational factor in shaping the gut microbiota, influencing its composition, diversity, and functionality, which in turn affects a wide range of health outcomes through complex microbial-host interactions. Diets rich in fiber, including those based on fruits, vegetables, legumes, and whole grains, act as prebiotics that nourish beneficial bacteria like *Bifidobacteria and Lactobacilli*. These bacteria produce SCFAs, such as butyrate, acetate, and propionate, which are essential for maintaining intestinal barrier integrity [26], regulating immune responses [27], and providing energy for colon cells, thus playing a protective role against metabolic disorders, colorectal cancer, and inflammatory bowel diseases (IBD) [28]. High-fiber diets have been shown to increase microbial diversity, an indicator of a resilient and healthy microbiome, which correlates with reduced risks of obesity, type 2 diabetes [29], and cardiovascular diseases [30,31]. Furthermore, dietary patterns like the Mediterranean diet (MD) have been found to promote a microbiome profile with anti-inflammatory effects, improved lipid metabolism [32], and enhanced cognitive function [33]. Conversely, Western dietary patterns, high in refined sugars, animal fats, and low in fiber, are associated with dysbiosis-a microbial imbalance marked by decreased diversity and increased populations of pathogenic bacteria like Bacteroides and Alistipes, which promote systemic inflammation [34], increase gut permeability [35], and contribute to conditions like insulin resistance and non-alcoholic fatty liver disease [36].

Specific dietary components exert unique effects on the gut microbiota. Polyphenols, found in plant-based foods like berries, tea, and olive oil, possess antioxidant and anti-inflammatory properties that benefit the gut microbiota by supporting the growth of beneficial bacteria [37] while inhibiting harmful species [38]. Similarly, plant-based proteins in legumes and nuts encourage the growth of health-promoting microbes [39], whereas a high intake of animal protein is associated with bile-tolerant bacteria such as Bilophila, which are linked to inflammation and increased gut permeability [40]. Soluble fibers, abundant in foods like oats, apples, and citrus fruits, not only serve as a food source for gut bacteria but also play a role in reducing harmful bacterial adhesion to the gut lining, thus supporting the gut barrier [41] and enhancing immune function [42]. In contrast, diets high in processed foods can lead to the production of harmful metabolites like trimethylamine-N-oxide (TMAO), which is associated with cardiovascular disease risks [43]. Research underscores the therapeutic potential of dietary interventions targeting the microbiome. Plant-based diets, such as the Mediterranean, vegetarian, and other fiber-rich diets, have been associated with enhanced microbial diversity [44] and SCFA production, offering a non-invasive approach to managing dysbiosis and reducing the risk of inflammatory and metabolic diseases [45]. These insights highlight the significant role of diet in modulating gut health and the potential for dietary interventions as preventive and therapeutic strategies for promoting a healthy gut microbiome and overall wellness.

Dietary interventions and therapeutic applications for mental health

Diet is a major factor influencing gut microbiota and, subsequently, mental health. Diets high in fiber, prebiotics, and probiotics promote beneficial bacterial growth, enhance SCFA production, and reduce inflammation, all of which collectively support resilience against stress and improve cognitive function [46,47]. Conversely, Western diets rich in processed foods, refined sugars, and unhealthy fats have been shown to reduce microbial diversity, increase gut permeability, and heighten systemic inflammation, which can exacerbate symptoms of mood disorders [48,49]. Such diets can also diminish populations of beneficial bacteria, reducing SCFA production and promoting the growth of pro-inflammatory bacterial species [50,51]. Chronic stress further influences the GBA by decreasing beneficial bacterial populations and increasing those associated with inflammation, raising susceptibility to neuropsychiatric conditions such as anxiety and depression [52-55].

Nutritional interventions such as diets rich in antioxidants like vitamins C and E, polyphenols, and omega-3 fatty acids have shown promise in alleviating mental health symptoms by enhancing microbial diversity and fortifying the gut-brain connection [56-58]. The MD, in particular, has been associated with increased microbial diversity and the production of beneficial metabolites, which support cognitive resilience and emotional regulation [59]. Similarly, plant-based diets, high in fiber and prebiotics, foster a gut environment that supports mental well-being through enriched microbial diversity and elevated SCFA production [60,61].

The immune system is another critical player in the GBA, with gut bacteria influencing immune responses that can affect the CNS. An imbalanced microbiome often promotes pro-inflammatory cytokine production, which increases neuroinflammation and can impair cognitive and emotional regulation [62]. Tryptophan, a precursor to serotonin, is heavily influenced by gut microbiota [62]. Disruptions in tryptophan metabolism due to dysbiosis have been linked to depressive symptoms [63]. Advanced therapeutic approaches, such as combining dietary modifications with psychobiotic supplementation, offer promising strategies for managing mental health disorders by supporting microbial diversity, reducing inflammation, and strengthening the GBA [64,65]. As this field evolves, personalized nutritional and microbiome-focused strategies are increasingly being explored as tools for improving emotional resilience and cognitive function. While still an area of ongoing investigation, dietary patterns that promote a healthy gut microbiome may hold therapeutic potential in mental health care.

Impacts of specific diets on gut health

Impact of Mediterranean Diet on Gut Health

The MD has a profound impact on gut health due to its nutrient-rich, plant-based composition, which promotes a favorable gut microbiota profile linked to various health benefits. High in fiber, polyphenols, and unsaturated fats from sources such as olive oil, fruits, vegetables, whole grains, nuts, and legumes, the MD supports microbial diversity [66] and enhances the abundance of beneficial bacteria like *Bifidobacteria, Lactobacillus, Faecalibacterium prausnitzii, and Bacteroides* [67]. As described earlier, these bacteria produce SCFAs that help maintain gut barrier integrity [68], reduce inflammation [69], and support immune health [70]. Studies indicate that adherence to the MD correlates with higher microbial diversity, which is essential for metabolic resilience and gut health. For example, a systematic review of randomized controlled trials found that adherence to the MD increases SCFA-producing bacteria, which play a role in reducing gut permeability and systemic inflammation, thereby potentially mitigating the risk of chronic inflammatory diseases [67,70,71]. Additionally, the MD’s high fiber and polyphenol content supports the growth of anti-inflammatory bacteria while inhibiting pathogens, contributing to a gut environment that lowers the risk of gastrointestinal disorders [68], cardiovascular diseases [72], and metabolic syndrome [73].

Controlled studies comparing the MD with Western diets high in saturated fats and refined sugars suggest that MD adherence may promote greater bacterial diversity and an increased abundance of beneficial bacteria. Some studies have also associated higher intake of plant-based foods with a more favorable *Bifidobacteria: E. coli* ratio, potentially contributing to reduced gut inflammation [71,74]. Furthermore, the inclusion of monounsaturated fats, particularly from olive oil, has been shown to support microbial communities that produce anti-inflammatory compounds, underscoring the protective role of the MD on gut health [66,74]. The MD also promotes a healthier microbiome by encouraging populations that produce beneficial metabolites while limiting those linked to metabolic such as SCFAs like butyrate and acetate, and cardiovascular diseases, such as TMAO [74,75]. Overall, the MD's focus on whole foods, plant-based sources of protein, and unsaturated fats offers a non-invasive dietary approach to supporting gut health and reducing the risk of a range of inflammatory and metabolic conditions through microbiome modulation [72,65].

Impact of Plant-Based Diet on Gut Health

A plant-based diet has a notable impact on gut health due to its high fiber, polyphenol, and phytonutrient content, which support beneficial bacterial populations and promote a balanced gut ecosystem. Plant-based diets, including vegetarian and vegan diets, are associated with increased microbial diversity, a critical marker for gut resilience and overall health [75]. Fiber-rich foods, such as fruits, vegetables, legumes, and whole grains, act as prebiotics, encouraging the growth of fiber-fermenting bacteria like Bifidobacterium, Prevotella, and Bacteroides [76]. These bacteria ferment dietary fibers to produce SCFAs, such as acetate, propionate, and butyrate, which are essential for gut health. SCFAs provide energy to colon cells [77], regulate immune responses [78], and strengthen the gut barrier, reducing gut permeability and potentially protecting against conditions like irritable bowel syndrome (IBS) and IBD [79].

Additionally, polyphenols in plant-based foods provide antioxidant and anti-inflammatory effects, which favorably influence gut microbiota composition by promoting the growth of beneficial bacteria and inhibiting pathogens [80]. This microbial balance creates an environment that reduces the risk of inflammation, both within the gut and systemically [81]. Vegan and vegetarian diets, in particular, have been linked to lower levels of pathobionts, such as Enterobacteriaceae, which are associated with gut inflammation [78]. Furthermore, the higher intake of fiber and polyphenol-rich foods in plant-based diets is associated with an increased abundance of Bacteroidetes relative to Firmicutes, a balance that has been correlated with a leaner body composition and a reduced risk of metabolic disorders like obesity and type 2 diabetes [79,80]. Regular consumption of plant-based diets has also been linked to cardiovascular health benefits, including reductions in LDL cholesterol and blood pressure, partly due to microbiota-mediated pathways. These diets reduce the production of TMAO [81]. TMAO is a metabolite produced by certain gut bacteria linked to heart disease [82]. Together, these findings highlight the potential of plant-based diets to improve gut microbiota diversity and function, offering broad health benefits from metabolic stability to reduced chronic disease risk through positive modulation of the gut microbiome.

Emerging evidence suggests that gluten, particularly in individuals with non-celiac gluten sensitivity (NCGS) or celiac disease, may influence mental health via increased intestinal permeability, immune activation, and subsequent neuroinflammation. Gluten-induced gut dysbiosis and systemic inflammation have been associated with mood disturbances such as anxiety, depression, and cognitive dysfunction [83].

Key nutrients and their impact on mental health

Impact of Omega-3 Fatty Acids on Mental Health

Omega-3 fatty acids, particularly eicosapentaenoic acid (EPA) and docosahexaenoic acid (DHA), are critical components of neural membranes, influencing brain function and mental health. These fatty acids demonstrate significant anti-inflammatory properties, making them essential in mitigating the neuroinflammation associated with mental health conditions like depression. Studies have shown that EPA reduces depressive symptoms by modulating cytokine production and enhancing neurotransmitter function [84]. Supplementation with omega-3s has been especially effective in individuals with major depressive disorder, though responses may vary based on individual metabolic differences and dosage [85].

Anxiety and stress-related disorders are also impacted by omega-3 levels. Clinical trials have indicated that supplementation reduces anxiety symptoms in healthy adults, particularly those under high stress, such as medical students. This reduction is attributed to a decrease in pro-inflammatory cytokines like interleukin-6 (IL-6), which are often elevated in stress-related conditions. Moreover, populations with lower omega-3 levels have demonstrated heightened anxiety and reduced stress resilience, underscoring the fatty acid’s critical role in emotional regulation [86,87].

The role of omega-3s extends to cognitive decline and neurodegenerative disorders. Omega-3 fatty acids protect against diseases like Alzheimer’s and Parkinson’s by reducing oxidative stress, decreasing neuroinflammation, and supporting neuronal membrane integrity [88]. Although evidence suggests that omega-3 supplementation can delay cognitive decline in early Alzheimer’s stages, its effects in advanced stages remain less consistent [88-90]. In children with ADHD, omega-3 supplementation has improved attention and cognitive performance [91]. This reflects the fatty acid's impact on neurotransmitter pathways and neural development [92]. Similarly, in autism spectrum disorders (ASD), omega-3 supplementation shows potential in reducing neuroinflammation and behavioral symptoms, although more research is needed to confirm these benefits [93].

Mechanistically, omega-3 fatty acids exhibit their effects through several pathways. They regulate neurotransmitter systems, particularly dopaminergic and serotonergic pathways, which are crucial for mood and cognitive functions [89,90]. Additionally, DHA enhances membrane fluidity and synaptic plasticity [88]. This facilitates effective neuronal signaling and cognitive processes [92]. The anti-inflammatory properties of omega-3s, achieved by competing with pro-inflammatory omega-6 eicosanoids, further underline their role in preventing chronic inflammation-related mental health disorders [84,89]

Effective supplementation for mental health benefits often involves daily doses of 1 to 3 grams of EPA and DHA, with EPA demonstrating superior efficacy in reducing depressive symptoms [86,90]. The high omega-6 to omega-3 ratio in Western diets exacerbates the need for omega-3 supplementation, as this imbalance has been linked to increased prevalence of depression and other mood disorders [90].

Impact of Probiotics and Prebiotics on Mental Health

Probiotics and prebiotics, often referred to as psychobiotics when used to influence mental health, have garnered significant attention for their role in modulating the GBA. The GBA communicates through hormonal, neural, and immune pathways. Research suggests that probiotics, particularly strains like *Lactobacillus *and* Bifidobacterium*, may alleviate mental health conditions such as depression and anxiety by reducing systemic inflammation, modulating the HPA axis, and restoring gut microbiome balance [94]. Clinical trials have demonstrated significant reductions in depressive and anxiety symptoms, as well as lower levels of inflammatory markers such as IL-6, in individuals who supplemented with probiotics [95]. However, not all studies are consistent, with some reporting limited or no significant effects [96].

Probiotics and prebiotics also show potential in improving cognitive function. Probiotic supplementation has been linked to enhanced cognitive flexibility and reduced stress levels, particularly in older adults. These effects are often associated with shifts in gut microbiota composition and increased brain-derived neurotrophic factor (BDNF), a key protein for neuroplasticity [97]. Prebiotics, on the other hand, promote the production of SCFAs, which enhance gut barrier integrity and modulate neurotransmitter levels, contributing to better cognitive and emotional regulation [98]. Moreover, specific probiotic formulations like NVP-1704 (*Lactobacillus reuteri* NK33 and *Bifidobacterium adolescentis *NK98) have shown efficacy in improving sleep quality and reducing stress in healthy adults, further underscoring the gut microbiota’s role in mental well-being [99].

While strain specificity plays a key role in probiotic efficacy, dosage is also important. For example, a randomized, double-blind, placebo-controlled trial found that daily supplementation with NVP-1704 (containing 2.5 × 10⁹ CFU of *Lactobacillus reuteri* NK33 and *Bifidobacterium adolescentis* NK98) for 8 weeks significantly improved subclinical symptoms of depression, anxiety, and insomnia in healthy adults, likely via gut microbiota modulation and reduced systemic inflammation [95]. However, given the heterogeneity in study populations and strains used, no universal dosing guidelines currently exist, emphasizing the need for further dose-response research.

The relationship between the gut microbiome and neurodevelopmental conditions, such as ASD, has also been explored. Although some studies report improvements in gastrointestinal symptoms and behavioral outcomes in children with ASD following probiotic or prebiotic interventions, others find limited or inconsistent results, highlighting the need for further research [100]. Similarly, while preliminary findings suggest that psychobiotics could offer benefits for maternal mental health and stress reduction during pregnancy, evidence remains inconclusive and requires more robust studies [101].

Mechanistically, probiotics and prebiotics influence mental health by modulating the production of neurotransmitters such as serotonin and GABA, as well as reducing neuroinflammation. These compounds also improve the gut barrier and immune function, creating a favorable environment for GBA communication [94]. Despite the promising findings, the efficacy of psychobiotics is influenced by various factors, including the specific strains, dosages, and individual microbiome composition, necessitating personalized approaches to treatment [95]. Overall, probiotics and prebiotics hold substantial promise as adjunct therapies for mental health, offering a non-invasive, cost-effective approach that could complement traditional psychiatric interventions, especially for individuals with limited access to mental health care [96-99].

Impact of Antioxidants on Mental Health

Antioxidants play a vital role in maintaining mental health by mitigating oxidative stress, a key factor in the pathogenesis of neurodegenerative and psychiatric disorders. The brain is particularly susceptible to oxidative damage due to its high oxygen consumption, lipid-rich composition, and limited antioxidant defenses. Research has demonstrated that antioxidants such as glutathione, vitamin C (ascorbate), and vitamin E (α-tocopherol) protect neurons by scavenging reactive oxygen species (ROS) and reducing oxidative damage to DNA, lipids, and proteins [101,102]. These neuroprotective effects are especially significant in conditions like Alzheimer’s and Parkinson’s diseases [103], where oxidative stress contributes to disease progression [104].

In psychiatric disorders such as depression, anxiety, and bipolar disorder, oxidative stress exacerbates symptoms, while antioxidant supplementation has been shown to improve outcomes. Clinical studies indicate that individuals with generalized anxiety disorder (GAD) and depression have significantly lower levels of antioxidants in their systems. Supplementation with antioxidants such as β-carotene, polyphenols, and vitamins A, C, and E has been associated with reduced symptoms of depression and anxiety, as well as improved stress resilience [105,106]. Moreover, dietary antioxidants have shown an inverse relationship with psychiatric disorders, suggesting that diets rich in fruits, vegetables, and other antioxidant sources can serve as preventive measures against mental health deterioration [107,108].

The mechanisms by which antioxidants influence mental health involve the regulation of oxidative stress, inflammation, and mitochondrial function. By neutralizing ROS and reactive nitrogen species (RNS), antioxidants prevent cellular damage and support synaptic plasticity, which is critical for cognitive and emotional stability. Furthermore, they modulate redox-sensitive signaling pathways and transcription factors such as NF-κB, which are implicated in stress responses and inflammation [103,104]. These effects also extend to neuroprotection, where antioxidants like resveratrol and curcumin have shown potential in managing neurodegenerative and psychiatric conditions through their anti-inflammatory and free radical scavenging properties [104,107].

Dietary patterns rich in antioxidants, such as the MD, have been associated with improved mental health outcomes. These diets emphasize the consumption of antioxidant-rich foods like fruits, vegetables, nuts, and whole grains, which have been linked to lower risks of depression and cognitive decline. Epidemiological studies suggest that higher dietary antioxidant indices correlate with reduced stress, anxiety, and depressive symptoms, underscoring the importance of nutrition in mental health [108,109]. For instance, the Composite Dietary Antioxidant Index (CDAI) has shown a negative non-linear association with depression, highlighting the benefits of antioxidant-rich diets in reducing the risk of mental disorders [110].

## Conclusions

The complex connection between the GBA and mental health highlights how important dietary decisions are for emotional resilience, cognitive function, and general well-being. This review emphasizes the important impact of dietary changes on the gut microbiome, which affects processes like inflammation, neurotransmitter production, and neural signaling. Diets rich in fiber, plants, and Mediterranean influences, as well as nutrients such as probiotics, prebiotics, omega-3 fatty acids, and antioxidants, show promise in promoting microbial diversity and improving communication between the gut and brain, providing effective ways to alleviate symptoms of mental health conditions. Nevertheless, there are significant discrepancies in implementing these understandings into practical application. Contemporary research is frequently constrained by limited sample sizes, brief time frames, and a dearth of individualized methodology. It is important to comprehend the differences in microbiome makeup and reactions to dietary shifts to improve the effectiveness of these interventions. Customized nutrition, driven by progress in microbiome studies and data analysis, has the potential to transform the way diet treatments are personalized for each person.

Future studies should involve extended, multicenter clinical trials incorporating demographic, genetic, and lifestyle elements. Nutritionists, microbiologists, and mental health professionals need to work together to create comprehensive models of care. Additionally, public health programs must prioritize educating societies about the significance of GI health, increasing the availability of nutritious foods, and tackling challenges related to food insecurity and economic inequalities. Through utilizing diet and microbiome science, we can lead the way in developing creative methods for mental health treatment, providing potential for lasting and comprehensive resolutions to key issues in contemporary healthcare. The opportunity to enhance mental health with specific dietary techniques is a thrilling new area that connects nutritional research with mental well-being.
